# Validated analytical workflow for quantifying proline metabolism related amino acids in HaCaT cells with LC-Q-TOF-MS

**DOI:** 10.1038/s41598-026-58884-2

**Published:** 2026-06-22

**Authors:** Szymon Plewa, Magdalena Nizioł, Katarzyna Bielawska, Agnieszka Klupczynska-Gabryszak, Jan Matysiak, Wojciech Miltyk

**Affiliations:** 1 Department of Inorganic and Analytical Chemistry, Poznan University of Medical Sciences, 3Rokietnicka Street, 60-806 Poznan, Poland; 2 Department of Analysis and Bioanalysis of Medicines, Medical University of Bialystok, Bialystok, 15-222 Poland

**Keywords:** Metabolomics, Cell culture, Proline, Amino acids, Liquid chromatography-mass spectrometry, Biochemistry, Biological techniques, Chemistry

## Abstract

It is known that altered amino acid metabolism can influence immunological responses, thus studying the molecular mechanisms underlying these changes requires analytical methods for reliably determining the quantities of individual analytes. To address the ongoing need for quantitative assessment of amino acid composition, a validated method for determining their quantities was proposed. This study presents the development and validation of a novel method for the quantitative analysis of selected amino acids using high-performance liquid chromatography coupled with a quadrupole time-of-flight (Q-TOF) mass spectrometer equipped with an electrospray ionization source. The method has been satisfactorily validated in terms of linearity, range, precision, accuracy, and stability. Its applicability to biological matrices was demonstrated by the successful quantification of amino acids in HaCaT cell lysates. Owing to the use of a robust, state-of-the-art analytical platform based on Q-TOF technology, which enables high-resolution mass detection, the method is readily adaptable for the inclusion of additional analytes and can be extended to other complex biological samples. The applicability of the method for the simultaneous determination of selected amino acids was demonstrated, and the method successfully applied to the HaCaT cell lysates.

**Supplementary Information:**

The online version contains supplementary material available at https://doi.org/10.1038/s41598-026-58884-2.

## Introduction

Skin constitutes the body’s integral protective envelope and plays a key role in protecting internal homeostasis from external factors. Disruption of skin integrity or its homeostasis at the molecular level (e.g., inflammation) requires appropriate treatment, hence the growing interest in research on the molecular basis of skin homeostasis^1,2^. Previous studies have postulated a role of proline metabolism in inflammation and wound healing^3–5^. Nevertheless, to investigate the molecular background processes underlying these conditions, the quantitation of appropriate intermediates was needed. Proline appears to link many biological mechanisms, with collagen turnover and the related proline cycle of particular interest. Recently, proline-driven intracellular signaling, as well as apoptosis and autophagy, have played an emerging role in the understanding of cellular metabolism^5,6^. Biochemically, proline originates from the enzymatic conversion of pyrroline-5-carboxylic acid (P5C), which can derive from either glutamate or ornithine^7^, indicating that the proline cycle remains in a relationship with the tricarboxylic acid (TCA) and urea cycle. The alternative source of free proline is collagen turnover, providing proline and hydroxyproline, which together account for about one-fourth of the total amino acid content in the protein^8^. Thus, proline and its metabolic companions are considered key contributors to wound healing^9^. The explanation of the biological effects of prolidase on intracellular pathways involved in the crucial processes of tissue remodeling and cell proliferation common during the inflammatory stage of skin wound healing was recently reported^3^. One of our emerging interests was to study the effect of recombinant human PEPD (rhPEPD) wild-type (WT) and mutated forms of rhPEPD on the selected amino acids linked to proline metabolism in an experimental model of inflammation in IL-1β-treated HaCaT keratinocytes.

Several analytical strategies have been reported for amino acid quantification in biological matrices. Classical amino acid analyzers based on ion-exchange chromatography (IEC) with post-column ninhydrin derivatization provide comprehensive amino acid profiling but are time-consuming (typically > 60 min) and offer limited sensitivity. Pre-column derivatization strategies employing ortho-phthalaldehyde (OPA), fluorenylmethyloxycarbonyl chloride (FMOC), or 6-aminoquinolyl-N-hydroxysuccinimidyl carbamate (AccQ-Tag), coupled with reversed-phase LC with mass spectrometric or fluorescence detection, have been widely adopted due to their excellent sensitivity^10–12^. However, derivatization introduces additional analytical steps that increase variability and require strict reaction control. Hydrophilic interaction liquid chromatography (HILIC) enables direct separation of underivatized amino acids by exploiting their inherent polarity, and coupling HILIC with high-resolution mass spectrometry (HRMS) provides accurate mass measurements that ensure high selectivity without derivatization. Moreover the HILIC-Q-TOF platform allows straightforward addition of new analytes without re-optimizing derivatization conditions, supporting the method’s intended future expandability. Targeted LC-HRMS methods for amino acid quantification in cell cultures have been previously described^13–15^, but methods specifically tailored to the proline metabolic network and fully validated for cell lysates remain limited.

Further research was required to develop a quantitative analysis tool for measuring proline and other amino acids involved, e.g., in proline turnover. The first step to overcome this obstacle was to develop a method for the quantitation of selected amino acids previously proposed by us^9^. However, there was an interest in the scientific community to expand the panel of measured amino acids. Therefore, ongoing efforts have focused on broadening the spectrum of measured amino acids using a targeted approach. Based on a previous study, a new method was developed for the quantification of 8 amino acids. To use the technological advantages of contemporary analytical instruments a high-performance liquid chromatograph (HPLC) coupled to a quadrupole-time of flight (Q-TOF) mass spectrometer was used. It was very important to us to propose a high-throughput, robust method, which could be easily modified, and after revalidation, applied to other matrices, and if necessary, extended by additional compounds of interest. The satisfactory results of validation, and application of the newly developed method for quantitation of the panel of amino acids in cell lines proved that it could be a competent alternative to the gold standard of targeted analysis, i.e., triple quadrupole mass spectrometers (QqQ). As we showed, satisfactory validation results and the possibility to further expand the spectrum of analytes while maintaining accurate masses ensure the usefulness of the proposed methodology for a wide range of new applications.

## Materials and methods

### Reagents

Deionized water (18.2 MΩ-cm resistivity at 25 °C) was obtained from the Milli-Q^®^ Advantage A10 water purifying system (Merck Millipore, Darmstadt, Germany). The following amino acid reference standards were used: L-Proline (cat. no. 81709, ≥ 99.5% (NT)), L-Arginine (cat. no. 11009, ≥ 99.5% (NT)), L-Ornithine monohydrochloride (cat. no. 57197, ≥ 99% (TLC)), L-Glutamic acid (cat. no. PHR1107, 99.9%), L-Glutamine (cat. no. 49419, ≥ 99.5% (NT)), L-Alanine (cat. no. 05130, ≥ 99.0% (NT)), Glycine (cat. no. G7126 ≥ 99% (HPLC)), trans-4-Hydroxy-L-proline (cat. no. 41875, ≥ 99% (TLC)), and L-Proline-2,5,5-d_3_ (stable isotope-labeled internal standard, cat. no. D-6266, 98 atom% D). The liquid chromatography-mass spectrometry (LC-MS) grade solvents: acetonitrile, methanol, isopropanol and mobile phase modifier: formic acid, ammonium formate were obtained from Sigma-Aldrich (St. Louis, MO, USA). The reference mass solution kit and tuning mix for Q-TOF-MS calibration were purchased from Agilent Technologies (Santa Clara, CA, USA).

### Preparation of standard solutions and calibrators

Stock solutions of analytes were prepared at two concentrations (c = 0.5 M and c = 0.05 M) in water and stored at 4 °C. Due to the limited solubility of glutamic acid and L-glutamine in water, these solutions were prepared at a lower concentration (c = 0.05 M). The rest of the stock solutions were at the same concentration (c = 0.5 M). Working solutions of the compounds of interest at three concentrations (c = 5 mM, 0.5 mM, and 0.05 mM) were prepared by mixing appropriate volumes of stock solutions and diluting the resulting mixture in methanol. Stock solutions of IS were prepared by accurately weighing proline-d_3_ and dissolving it in 1.0 mL of water (c = 0.05 M). A working solution of IS was prepared by diluting the stock solution in LC-MS grade methanol (c = 1.25 mM). Calibrators were prepared by mixing appropriate working solutions of compounds of interest with IS working solution and diluting with methanol. Methanol was selected as the calibrator diluent because the quantified amino acids are endogenous compounds for which a genuinely analyte-free biological matrix cannot be obtained; methanol is fully compatible with HILIC retention conditions and ensures consistent chromatographic behavior and is also readily used in metabolomics studies for quenching and metabolite extraction, or sample resuspension prior mass spectrometry analysis^9,16^. Matrix effects were assessed independently using a BSA/PBS surrogate matrix (1 mg/mL BSA), as described in the Method Validation section.

### Cell cultures sample preparation

HaCaT spontaneously immortalized human keratinocytes (Cell Line Service GmbH; Eppelheim, Germany) were cultured in DMEM cell culture medium (PanBiotech, Aidenbach, Germany) with 10% fetal bovine serum (FBS; Gibco, Waltham, MA, USA) and 1% Penicillin/Streptomycin (Gibco, Waltham, MA, USA) in a cell incubator (37 °C, 5% CO2). For LC-MS-based analysis of the selected amino acids, cells were seeded at 2 × 10^5^ cells/well in 6-well plates. Once HaCaT cells reached 80% of confluency, cells were treated with human recombinant IL-1β (10 ng/ml; Sigma Aldrich, Saint Louis, MO, United States) and human recombinant prolidase (rhPEPDWT, rhPEPD-G448R, rhPEPD-231delY, and rhPEPD-E412K)^17^ at concentrations of 50 nM for 24 h. Cells subjected to PBS served as a control.

### Instrumentation for liquid chromatography–mass spectrometry

The assays were performed by means of a high-performance liquid chromatography system coupled to an electrospray ionisation quadrupole time of flight (Q-TOF) mass spectrometer platform. Chromatographic analysis was performed using a 1260 Infinity high-performance liquid chromatograph (Agilent Technologies, Santa Clara, CA, USA). Amino acids separation was carried out using a HPLC column InfinityLab Poroshell 120 HILIC-Z 2.1 × 100 mm, 2.7-micron (Agilent Technologies, Santa Clara, CA, USA), at 30 °C. The total run time was 9 min (Fig. S1). The elution was performed at the flow rate of 400 µL/min, under gradient conditions as follows: 0–6 min linear from 100% B to 30% solvent B, 6–7 min from 30% solvent B to 100% solvent B, 7–9 min holding 100% B, where solvent A contains 10% 20mM ammonium formate (NH_4_FA) in water, and solvent B contains 10% 20 mM NH_4_FA in acetonitrile (ACN). Injection volume was set at 1 µL with flushing the needle after each injection for 10 s using a mixture of isopropanol (IPA): water (1:1). The autosampler tray was maintained at 10 °C.

Detection was performed by means of a 6530 Q-TOF mass spectrometer with dual electrospray (dual-ESI) ion source controlled with MassHunter WorkStation software (Agilent Technologies, Santa Clara, CA, USA). The mass spectrometer was operated in positive-ion mode and full-scan mode over the range 50.0–1000.0 *m/z*. The operating dual-ESI source parameters were as follows: drying gas (N_2_) flow, 7 L/min; drying gas temperature, 300 °C; nebuliser gas, 40 psig; skimmer voltage, 65 V; capillary voltage, 3.0 kV. To correct small mass drifts at the time of acquisition and to ensure accurate mass calibration and system performance verification during all analyses, two reference masses at *m/z* 121.0509 and *m/z* 922.0098 were simultaneously introduced along with the LC stream. These reference masses correspond to protonated purine (*m/z* 121.0509) and protonated hexakis(1 H,1 H,3 H-tetrafluoropropoxy)phosphazene (HP-0921, *m/z* 922.0098), as provided in the Agilent reference mass solution kit (Agilent Technologies, Santa Clara, CA, USA). Data were collected in centroid mode at a rate of 1 spectrum per second. The data were acquired using the Extended Dynamic Range mode (2 GHz) and Agilent MassHunter Acquisition software (Agilent Technologies, Santa Clara, CA, USA).

### Method development

During method development, our previous LC-MS method underwent significant modifications^9^. For increasing the number of analytes, an alternative chromatographic column was proposed. The promising results of amino acid separation from standard solutions prompted a further step in method optimization. The different mobile phase composition, flow rates (0.3 mL/min and 0.4 mL/min), and various gradient conditions (in terms of the content of phase A in the mobile phase, and holding the constant phase composition during gradient conditions) were tested. During method development, the total runtime decreased from 16 min to 9 min in the presented method. The following ion source parameters were also tested to obtain the most satisfactory intensities of analytes (drying gas temperature: 275 °C, 300 °C, and 325 °C; capillary voltage: 2.5 kV and 3.0 kV). The selection of parameters such as flow rate, gradient conditions, and mobile phase compositions made it possible to propose an effective separation of eight amino acids in a single run under 9 min. The optimized parameters ensure fast and efficient separation of a panel of amino acids. In turn hyphenation with Q-TOF detection enables effective quantitation in a targeted manner, providing the finest mass accuracy (< 5 ppm), while also obtaining high specificity.

### Method validation

Linearity of the developed method was confirmed by analyzing calibration curves for each compound of interest using at least six non-zero calibrators, including the lower limit of quantification (LLOQ) in duplicate. Due to a lack of analyte-free biological matrices, calibrators were prepared by subsequent dilution of the standard solutions in LC-MS-grade methanol. Calibration curves were freshly prepared and analyzed directly prior to or after the QC samples. Each calibration curve was constructed using linear regression by plotting the peak area ratios of each metabolite to a proline-d_3_ as internal standards against its concentration. The LLOQ values were chosen as the lowest concentrations reliably determined from the background noise (signal-to-noise ratio for LLOQ), with satisfactory accuracy and precision (± 20% of the nominal value and relative standard deviation, respectively).

The carry-over was assessed by injecting a series of blank samples after the high calibrator sample. The analyte signal response lower than 20% of the signal from the LLOQ calibrator in the sample, followed by the calibrator high, was considered satisfactory.

### Accuracy, precision & recovery

Accuracy was evaluated by calculating the relative error (RE, bias) between the back-calculated concentration (from the calibration curve) and the nominal spiked concentration: RE (%) = [(calculated concentration − nominal concentration)/nominal concentration] × 100%. Evaluation of precision and accuracy was conducted by analyzing replicate QC samples at different concentration levels across the assay range. For accuracy and precision determination, the surrogate matrix samples containing BSA (bovine serum albumin) in PBS at a concentration of 1 mg/mL, spiked with standard solutions, were used. The freshly prepared QC samples were used for all accuracy and precision runs. The accuracy was evaluated by means of relative error (RE, bias), calculated according to the formula below:

$${\mathrm{RE}} = \left( {{\mathrm{calculated}}\;{\mathrm{value}} - {\mathrm{nominal}}\;{\mathrm{value}}} \right)/{\mathrm{nominal}}\;{\mathrm{value}} \times {\mathrm{1}}00\% .$$

Intrabatch and interbatch accuracy were assessed. QC samples at 4 concentration levels (LLOQ, low, medium, high) were analyzed in replicates, including a minimum of 3 independent runs. For intrabatch accuracy, 3 independent samples were analyzed in duplicate within one batch. For the interbatch accuracy assay, three batches, each with 3 independent samples injected in duplicate, were analyzed. The RE value within the ± 15% of nominal value (± 20% for LLOQ) was considered satisfactory.

Intrabatch and interbatch precision were tested by calculating % RSD of measured concentration analyzed QC samples at 4 concentration levels (LLOQ, low, medium, high) in replicates, including a minimum of 3 independent runs.

The recovery of the method was assessed by analyzing the additional set of QC samples, prepared by pooling and spiking the supernatants from metabolite extraction from cell lines, and calculated based on measured concentrations according to the formula:

$${\mathrm{Recovery}} = \left( {{\mathrm{spiked}} - {\mathrm{unspiked}}} \right)/{\mathrm{known}}\;{\mathrm{spike}} \times {\mathrm{1}}00\% .$$

Based on FDA recommendations, measured recovery was considered acceptable when it was consistent and reproducible. Additionally, interbatch and intrabatch % RSDs of the concentration were calculated to prove the precision of the analyte measurements. The measured precision parameters below 15% ( 20% for LLOQ) were considered satisfactory.

### Matrix effect

The matrix effect was evaluated by comparing the slopes of calibration curves prepared in neat methanol (methanol-based calibrators) with those prepared in a surrogate matrix (BSA in PBS, 1 mg/mL), and expressed as a percentage (Table 3). Matrix effect (%) was calculated as: Matrix Effect (%) = (slope in surrogate matrix/slope in neat methanol) × 100%. A value of 100% indicates no matrix effect; values > 100% reflect ion enhancement, and values < 100% reflect ion suppression. The slopes and coefficients of determination (r²) for all calibration curves used in this comparison are provided in the Supplementary Materials (Table S3).

### Stability

For method development, autosampler stability and freeze-thaw stability were evaluated. Given that the method is intended for the analysis of biological samples (cell lysates), a reduced autosampler temperature (10 °C) was adopted. Stability was assessed after 24 and 48 h for both unspiked pooled samples and calibrators at low and high concentration levels (Table S1). Both unspiked pooled samples and calibrators were analyzed in three replicates in 2 injections. The stability was calculated based on measured concentrations for analytes of interest according to the formula:

$${\mathrm{Stability}} = {\mathrm{measured}}\;{\mathrm{value}}\;{\mathrm{after}}\;{\mathrm{storage}}/{\mathrm{measured}}\;{\mathrm{value}}\;{\mathrm{for}}\;{\mathrm{freshly}}\;{\mathrm{prepared}}\;{\mathrm{sample}} \times {\mathrm{1}}00\% .$$

The acceptance criteria for stability testing was ± 15% of the nominal value, at each concentration level. Freeze-thaw stability was evaluated for unspiked pooled samples.

### Statistical analysis

All experiments were carried out at least in triplicates and the experiments were repeated twice. Data are shown as a mean ± standard deviation (SD). For statistical analysis, one-way analysis of variance (ANOVA) with Dunnett’s correction using GraphPad Prism 5.01 (GraphPad Software, Boston, Massachusetts USA) were performed. Statistically significant differences were marked as * *p* < 0.05, ** *p* < 0.01, *** *p* < 0.001.

## Results and discussion

### Specificity

Method specificity was ensured through the use of a Q-TOF— high resolution mass spectrometer by providing the finest mass accuracy, and the lack of signals in retention times of compounds of interest. Further, it was confirmed via analysing blank samples to exclude the presence of analytes, interferences, and impurities. Consistent retention times and sharp peaks, free of interferences or impurities at the retention times of analytes, demonstrated the method’s specificity. The high resolution Q-TOF instrument, provides accurate mass measurement effectively discriminating the target analytes from potentially interfering compounds with closely similar *m/z* values. No interfering signals were detected at the retention times of the eight target analytes, confirming adequate selectivity in the studied biological matrix.

**Table 1:** Method specification for eight amino acids compared with the concentrations found in HaCaT cell lines.

No	Compound	Abbreviation	Monoisotopic mass (Da)	m/z	Retention time*(min)	Method calibration range (µM)	Concentration range in HaCaT cell culture** (µM)
1	Alanine	Ala	89.047676	90.055	3.844	2.5–200.0	14.78–91.29
2	Arginine	Arg	174.111679	175.119	5.141	5.0–50.0	7.88–48.12
3	Glutamic acid	Glu	147.053162	148.0605	4.769	10.0-200.0	41.12–95.26
4	Glutamine	Gln	146.069138	147.0764	4.109	2.5–200.0	11.69–53.06
5	Glycine	Gly	75.032028	76.0393	4.059	25.0-200.0	11.44-176.68
6	Hydroxyproline	OH-Pro	131.058243	132.0656	3.847	10.0-200.0	9.75–51.28
7	Ornithine	Orn	132.089874	133.0972	5.371	5.0–50.0	< LLOQ
8	Proline	Pro	115.063332	116.0706	3.033	2.5–200.0	6.36–19.08

*average retention time of appropriate compound in real samples; **the range of concentrations for all injections of real samples.

Linearity & calibration range.

Due to the potential high dispersity of analyte concentrations in biological matrices (e.g., cell lines), during method development, we aimed to calibrate over a wide range of measured concentrations. In the results of performed pilot studies with cell lines, it was proposed the various calibration ranges for appropriate amino acids, as follows: proline 2.5–200, alanine 10.0-200.0, hydroxyproline 2.5–200.0; glycine 25.0–200.0, glutamine 2.5–200.0, glutamic acid 10.0–200.0, arginine 5.0–50.0, ornithine 5.0–50.0 µM (Table 1). The calibrators met the acceptance criteria when the final concentration of non-zero calibrators was ± 15% of the nominal concentration (± 20% for LLOQ). Linearity was achieved with correlation coefficients (r, weighting factor 1/X) between 0.9935 and 0.9989, providing evidence for satisfactory linearity of calibration.

### Carry-over

For ornithine the response in blank samples following the highest calibrator significantly exceeded the FDA bioanalytical method validation criterion (≤ 20%), reaching 82.50% of the LLOQ signal response (Table S2). This represents a significant limitation of the method in its present form. In the current application, ornithine was not detected above the LLOQ in any of the HaCaT cell lysate samples. Therefore, carry-over did not affect the reported results. Nonetheless, to ensure method robustness when applied to matrices where ornithine may be present at measurable concentrations, we recommend: (i) inclusion of at least two blank injections after each high-calibrator or high-concentration sample; (ii) implementation of extended automated needle wash cycles using IPA: H2O (1:1, v/v) after each injection; (iii) if ornithine concentrations are expected to be above the LLOQ in the target matrix, a dedicated carry-over assessment and, if necessary, additional chromatographic optimization should be performed prior to method application.

### Precision and accuracy

The precision, accuracy, and recovery of the newly developed method successfully met validation criteria (Tables 2 and 3).

**Table 2:** The accuracy and precision parameters of the method at four concentration levels of QC samples.

		Accuracy (%, bias)		Precision (%, RSD)	
	Level	intrabatch *n* = 3	interbatch *n* = 9	intrabatch *n* = 3	interbatch *n* = 9
Alanine	LLOQ	− 4.39	10.66	5.82	20.30
	LOW	7.27	14.32	4.06	11.26
	MED	6.02	8.32	3.41	12.79
	HIGH	6.54	10.44	5.08	6.38
Arginine	LLOQ	13.81	4.06	3.99	15.03
	LOW	11.90	5.77	1.84	7.42
	MED	3.09	2.53	1.61	5.35
	HIGH	− 10.65	− 8.92	1.63	7.38
Glutamic acid	LLOQ	17.67	10.70	2.61	13.51
	LOW	6.67	4.39	5.13	6.48
	MED	− 8.05	− 6.45	5.09	5.85
	HIGH	− 10.89	− 12.16	3.61	5.50
Glutamine	LLOQ	16.21	12.92	5.70	7.10
	LOW	9.76	8.90	3.71	4.75
	MED	11.16	2.52	2.37	7.80
	HIGH	− 13.64	− 16.63	2.33	4.73
Glycine	LLOQ	5.47	19.33	9.80	14.38
	LOW	9.74	7.42	5.02	6.14
	MED	11.65	4.62	1.84	8.45
	HIGH	12.58	12.21	2.15	4.36
Hydroxyproline	LLOQ	17.43	15.65	1.37	5.60
	LOW	− 4.52	− 3.87	5.62	4.76
	MED	− 13.37	− 12.32	3.70	3.14
	HIGH	8.25	4.43	3.95	5.14
Ornithine	LLOQ	17.75	10.48	2.06	6.66
	LOW	10.88	2.64	3.85	7.24
	MED	− 0.77	− 4.43	4.73	6.28
	HIGH	3.35	− 1.76	3.59	6.85
Proline	LLOQ	17.76	10.06	1.65	10.41
	LOW	6.32	6.45	0.90	3.98
	MED	8.32	7.41	1.84	4.82
	HIGH	12.66	10.45	1.71	5.06

### Recovery

**Table 3:** The recovery and precision parameters of the method at four concentration levels of the spiked sample.

Analyte	Level	Spike (µM)	Recovery		Precision (%, RSD)		Matrix effect (%)
			intrabatch *n* = 3	interbatch *n* = 9	intrabatch *n* = 3	interbatch *n* = 9	
Alanine	LLOQ	10.0	104.82	101.93	4.02	5.55	99.50
	LOW	30.0	105.61	99.72	5.77	6.75	
	MED	75.0	98.41	98.20	5.76	8.41	
	HIGH	150.0	94.75	95.56	8.18	8.46	
Arginine	LLOQ	5.0	103.60	101.57	8.55	8.11	134.13
	LOW	15.0	110.56	105.98	8.83	8.23	
	MED	25.0	112.85	108.80	7.98	7.62	
	HIGH	30.0	112.65	109.62	6.29	6.58	
Glutamic acid	LLOQ	10.0	103.19	105.60	4.23	11.30	140.40
	LOW	30.0	106.47	104.81	5.17	7.03	
	MED	75.0	110.42	105.62	4.55	8.03	
	HIGH	150.0	111.59	100.32	3.23	9.38	
Glutamine	LLOQ	2.5	103.67	99.33	6.04	7.31	127.83
	LOW	7.5	98.86	97.79	7.62	6.52	
	MED	50.0	91.27	92.93	8.60	7.89	
	HIGH	150.0	93.23	93.78	5.84	5.67	
Glycine	LLOQ	25.0	111.66	103.19	13.14	14.83	108.45
	LOW	75.0	108.41	101.75	15.07	14.38	
	MED	100.0	102.51	100.31	9.34	13.15	
	HIGH	150.0	109.34	105.56	5.98	13.37	
Hydroxyproline	LLOQ	2.5	109.08	87.95	5.12	5.61	97.62
	LOW	7.5	109.51	85.17	4.75	7.47	
	MED	50.0	72.86	63.78	6.65	16.98	
	HIGH	150.0	60.02	52.32	3.08	9.45	
Ornithine	LLOQ	5.0	108.80	99.54	17.37	15.85	121.28
	LOW	15.0	106.73	97.88	11.45	10.58	
	MED	25.0	110.03	100.85	11.10	12.03	
	HIGH	50.0	105.39	100.29	7.49	10.52	
Proline	LLOQ	2.5	98.96	92.72	6.42	4.57	115.19
	LOW	7.5	88.18	90.17	4.76	5.15	
	MED	50.0	105.18	100.11	8.53	11.32	
	HIGH	150.0	111.41	104.17	3.44	6.54	

### Matrix effect

The matrix effects ranged from 97.6 to 140.4% across the eight analytes (Table 3), with the highest ion enhancement observed for glutamic acid (140.40%), arginine (134.13%), and glutamine (127.83%). These values indicate that ionization enhancement of up to ~ 40% may occur for certain analytes in the BSA/PBS surrogate matrix. While the use of a single isotopically labeled IS (proline-d_3_) may not fully correct for matrix-induced signal changes for analytes with markedly different physicochemical properties, the recovery and accuracy data (Tables 2 and 3) demonstrate that the IS-normalized calibration yields quantitative results within accepted bioanalytical validation criteria. The use of analyte-specific stable-isotope-labeled standards for each quantified compound would represent the optimal approach and is recommended for future applications in matrices with higher complexity or where the most stringent accuracy requirements apply.

### Stability

The study proved the stability of the analytes under autosampler conditions (24 h and 48 h). All samples met the acceptance criteria for stability testing ± 15% of the nominal value, at both (low and high) concentration levels. The impact of three freeze-thaw (FT) cycles for compounds of interest was also assessed. Here, the effect of FT cycles has a stronger impact on measured analytes compared to short-term storage in the autosampler. For the selected chemical compounds, this effect exceeded 15% (Table S1) Therefore, we recommend not thawing samples intended for quantitative amino acid analysis in samples and performing analyses on fresh samples, if possible, or during the first thawing cycle.

The method was developed in response to the demand of the scientific community and the need for a fast and robust tool for quantitation of the spectrum of analytes in various matrices, including cell lines. The effectiveness of the proposed method was proven and confirmed by analysing the HaCaT cell lines that are the immortalized human keratinocytes. Until now, the HaCaT cell lines have been readily used to investigate the processes underlying skin homeostasis^18^. The aim of the presented research was not only to provide an analytical tool that can be successfully used for quantifying the amino acids concentration in e.g., cell lines, but also to apply the developed method to investigate the in vitro wound healing and skin inflammation model. Compared with previously described LC-MS methods for amino acid quantification in cell cultures, the present method offers several distinct features. Martano et al.^13^ described a fast LC-MS sampling method for mammalian cells using reversed-phase chromatography, but targeted a broader metabolomics panel without full bioanalytical validation of individual amino acids. Guitton et al.^14^ proposed an LC-HRMS method addressing matrix effects in cell culture samples. However, their approach focused on a different metabolite panel. Zhang et al.^15^ reported a UHPLC-HRMS method for 20 amino acids in lymphoma cells and serum. While broader in scope, their method was not specifically validated for proline metabolism intermediates in keratinocytes. In contrast, the present method provides a fully validated, targeted approach specifically optimized for the proline metabolic network, with calibration ranges adapted to HaCaT cell lysate concentrations, analysis time below 9 min, no derivatization requirement, and Q-TOF accurate mass measurement enabling future analyte expansion.

**Fig. 1:**
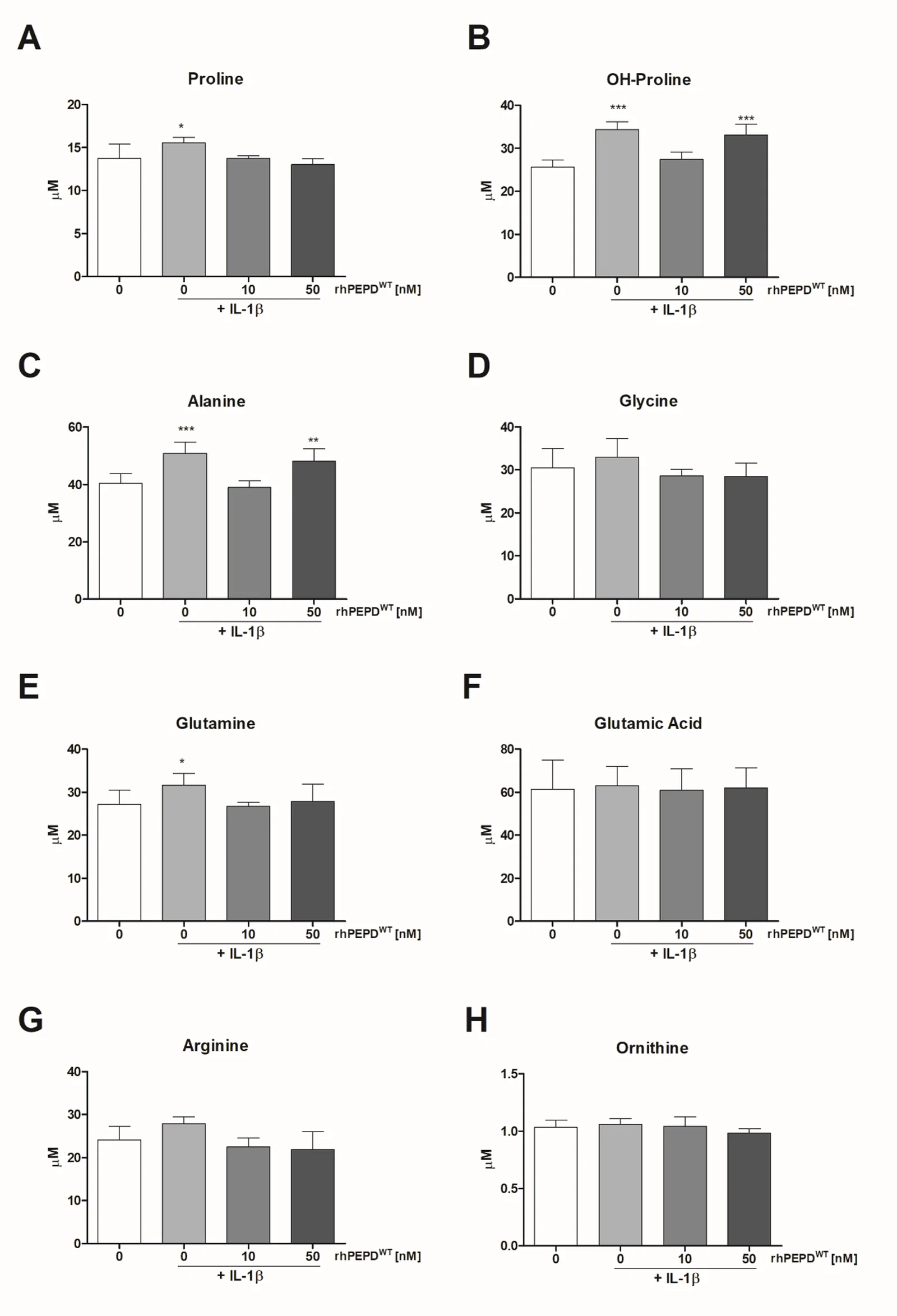
The intracellular effect of recombinant human prolidase (rhPEPDWT) in IL-1β-treated HaCaT keratinocytes on the selected amino acids (**A**) proline, (**B**) OH-proline, (**C**) alanine, (**D**) glycine, (**E**) glutamine, (**F**) glutamic acid, (**G**) arginine, and (**H**) ornithine. Statistical significances presented as * *p* < 0.05, ** *p* < 0.01, *** *p* < 0.001 are indicated versus non-treated HaCaT cells. The Figure was prepared using GraphPad Prism for Windows, GraphPad Software, Boston, Massachusetts USA, www.graphpad.com.

It has been proven that LC-MS-based targeted analysis of proline, glutamic acid, and arginine is useful in the in vitro model for wound healing^9^. In this study, our target metabolites, apart from the above-mentioned, are hydroxyproline, alanine, glycine, glutamine, and ornithine in the experimental model for inflammation-driven wound healing in the HaCaT cell line. It has been already demonstrated that prolidase, via activation of the epidermal growth factor receptor (EGFR), leads to stimulation of the anabolic signaling pathways under inflammatory conditions^3^. Since extracellular prolidase induces intracellular biological effects, we were interested in whether and how the metabolism of proline and related amino acids is involved. In the extracted HaCaT cell content, we observed that out of the eight target metabolites, seven were detected at measurable levels (above LLOQ). Figure 1 presents the results of the analysis showing that under inflammatory conditions, the metabolism of the selected amino acids is affected. The intracellular concentrations of proline, OH-proline, alanine, and glutamine were significantly increased in IL-1β-treated cells. When rhPEPDWT was present in the medium, proline and glutamine recovered to their starting levels, while OH-proline and alanine remained elevated compared with non-treated cells. In IL-1β-treated cells, the proline concentration equalled 15.54 ± 0.65 µM, while in untreated cells, it was 13.73 ± 1.68 µM. The intracellular concentration of glutamine significantly increased under IL-1β treatment (31.69 ± 2.76 µM) compared to control keratinocytes 27.25 ± 3.22 µM. The level of OH-proline rose from 25.70 ± 1.61 µM in control cell extract to 34.37 ± 1.79 µM in IL-1β-treated HaCaT cells, while treatment with IL-1β and rhPEPDWT (50 nM) showed 33.16 ± 2.49 µM. Similarly, the concentration of alanine in control was 40.47 ± 3.48 µM, while under inflammatory conditions, it increased to 50.98 ± 3.84 µM, and slightly decreased in rhPEPDWT-treated cells (50 nM) to 48.17 ± 4.25 µM.

**Fig. 2:**
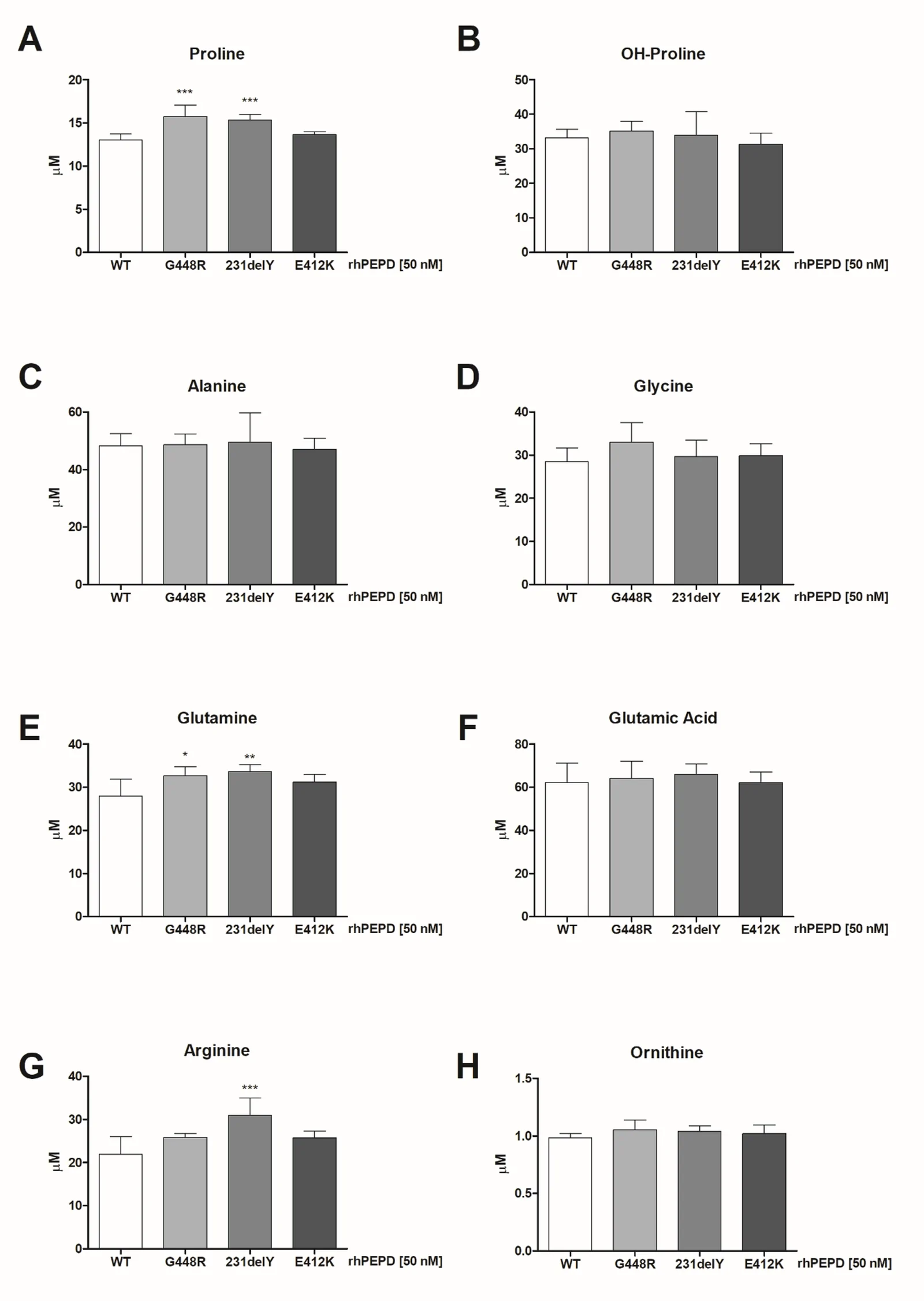
The intracellular effect of recombinant human prolidase forms(rhPEPDWT, rhPEPD-G448R, rhPEPD-231delY, and rhPEPD-E412K) in IL-1β-treated HaCaT keratinocytes on the selected amino acids (**A**) proline, (**B**) OH-proline, (**C**) alanine, (**D**) glycine, (**E**) glutamine, (**F**) glutamic acid, (**G**) arginine, and (**H**) ornithine. Statistical significances presented as * *p* < 0.05, ** *p* < 0.01, *** *p* < 0.001 are expressed versus rhPEPDWT-treated HaCaT cells. Figure was prepared using GraphPad Prism for Windows, GraphPad Software, Boston, Massachusetts, USA, www.graphpad.com.

As we showed above, wild-type prolidase was able to alter the intracellular metabolism of proline and its related amino acids under the studied conditions. Since in patients with prolidase deficiency (PD) skin ulcers are observed^19^, it was out of our curiosity to test whether mutated forms of this protein (rhPEPD-G448R, rhPEPD-231delY, and rhPEPD-E412K), naturally occurring in PD patients, would affect the analyzed metabolic pathways in the experimental model of inflammation. The IL-1β-treated HaCaT were treated with 50 nM of rhPEPD^WT^, rhPEPD-G448R, rhPEPD-231delY, and rhPEPD-E412K for 24 h (Fig. 2). We found that the intracellular concentrations of proline and glutamine were significantly elevated when treated with rhPEPD-G448R and rhPEPD-231delY in comparison to wild-type prolidase. In the case of the level of arginine, a similar effect was detected only in rhPEPD-231delY-treated cells. In IL-1β-treated HaCaT cells, the treatment with rhPEPD-G448R and rhPEPD-231delY increased the concentration of proline (15.73 ± 1.33 µM and 15.31 ± 0.67 µM, respectively) compared to rhPEPD^WT^ (13.04 ± 0.67 µM). Similar effects were observed with the same PEPD forms, which contributed to elevated glutamine levels. In rhPEPD-G448R-treated cells glutamine equalled 32.72 ± 2.04 µM, and in rhPEPD-231delY-treated cells 33.65 ± 1.60 µM, while in rhPEPD^WT^-treated keratinocytes, it was 27.92 ± 3.99 µM. The concentration of arginine rose from 21.95 ± 4.10 µM in rhPEPD^WT^-treated cells to 30.93 ± 4.09 µM after treatment with rhPEPD-231delY.

We are aware that the presented method has limitations, including a limited spectrum of quantified analytes and the use of a single stable-isotope-labeled internal standard (proline-d_3_) rather than individual labeled standards for each quantified compound. The use of analyte-specific stable-isotope-labeled internal standards (e.g., a commercially available labeled amino acid mix) would represent an improvement, particularly for analytes with physicochemical properties differing significantly from proline, and is recommended for future iterations of the method where the most stringent accuracy requirements apply. Nevertheless, the methodology is specifically tailored to the proline metabolism-related amino acids panel.

To meet the expectations of the scientific community, a new method of quantitation of a panel of proline-related amino acids was proposed. The method was validated, and it has proven to be useful for biomedical applications by analyzing the concentrations of selected amino acids in an experimental model of IL-1β-induced inflammation in human immortalized keratinocytes (HaCaT cell line). The optimized parameters ensure fast and efficient separation of eight amino acids, in turn, hyphenation with Q-TOF detection enables effective quantitation in a targeted manner, providing the finest mass accuracy (<5 ppm). The method was rigorously validated according to FDA bioanalytical method validation guidelines—including linearity, carry-over, accuracy, precision (intra- and interbatch), recovery, matrix effect, and stability and may serve as an excellent alternative to ready-to-use kits for the targeted determination of small-molecule compounds, demonstrating that high-resolution mass spectrometry (HRMS) instruments can meet validation criteria, allowing reliable qualitative results alongside accurate mass measurements. It should be noted that the current method, comprising eight amino acids of the proline metabolic network, is primarily suited for studies of proline turnover, collagen metabolism, and the interplay between proline, glutamine, and arginine. While alanine, glycine, proline, and glutamate have been associated with redox homeostasis and mitochondrial metabolism in the literature^20,21^, definitive conclusions regarding mitochondrial dysfunction or oxidative stress status would require a broader analyte panel and dedicated experimental validation. The potential of the method for such applications remains to be demonstrated in future studies. Nonetheless, a tailored, dedicated LC-MS method, optimized for the quantitation of proline metabolic pathway- related amino acids, would provide critical insights into proline metabolism, enable biomarker discovery, and support clinical diagnostics and therapeutic monitoring. Moreover, the developed method gives an excellent basis for expanding the spectrum of analytes that can serve as markers of biochemical changes in various biological matrices. Compared to previously described, derivatization-base methods for amino acids determination, the HILIC-Q-TOF platform allows straightforward addition of new analytes without re-optimizing derivatization conditions, supporting the method’s intended future expandability. The proposed analytical approach therefore integrates the robust quantitative performance typically associated with targeted triple quadrupole methodologies with the accurate mass measurement capabilities of high-resolution mass spectrometry (HRMS). In addition, the method offers convenient scalability and flexibility for the incorporation of newly emerging analytes, which constitutes a key advantage of high-resolution analytical platforms.

## Conclusions

A novel, validated HILIC-Q-TOF method for the simultaneous quantification of eight proline metabolism-related amino acids-proline, hydroxyproline, glutamic acid, glutamine, arginine, ornithine, alanine, and glycine—has been developed and successfully applied to complex biological matrices. The method was successfully validated with respect to linearity, intra- and inter-batch accuracy and precision, recovery, matrix effects, and stability. The total run time of under 9 min and the elimination of derivatization steps represent practical advantages over classical amino acid analyzers and pre-column derivatization approaches. Owing to the high-resolution accurate mass measurement capability of the Q-TOF platform, the method is readily expandable to additional analytes without re-optimization of derivatization chemistry, constituting a significant benefit for future metabolomics applications. Certain limitations of the current method should be acknowledged. The single stable-isotope-labeled internal standard (proline-d₃) may not fully compensate for matrix-related ionization effects for certain analytes. Additionally, the significant carry-over observed for ornithine represents a current constraint requiring additional chromatographic or injection-cycle optimization before the method can be reliably applied to matrices where ornithine concentrations exceed the LLOQ. The use of analyte-specific stable-isotope-labeled standards is recommended for future iterations where the most stringent accuracy requirements apply. Overall, the proposed methodology integrates the quantitative performance characteristic of targeted triple-quadrupole platforms with the accurate-mass and flexibility advantages of high-resolution mass spectrometry, providing a robust analytical framework for investigating dysregulation of the proline metabolic network in cell-based disease models and supporting further mechanistic and functional studies.

## Supplementary Information

Below is the link to the electronic supplementary material.

Supplementary Information 1

## Data Availability

The datasets used and/or analysed during the current study are available from the corresponding author on reasonable request.
