# Supplementary material for: Validated analytical workflow for quantifying proline metabolism related amino acids in HaCaT cells with LC-Q-TOF-MS
Source: Sci Rep. 2026 Jun 22;16:28563. doi: 10.1038/s41598-026-58884-2 (PMC13572413; doi:10.1038/s41598-026-58884-2)
Supplement: Supplementary file 1 [file 41598_2026_58884_MOESM1_ESM.docx]

**Supplementary materials.**

This document contains the following supplementary items:

**Table S1:** Autosampler stability (24 h and 48 h) and freeze-thaw stability data for all eight analytes at low and high QC concentration levels.

**Table S2:** Carry-over assessment results for all eight analytes following injection of the highest calibrator.

**Table S3:** Calibration curve slopes and coefficients of determination (r²) for calibration curves prepared in neat methanol and in surrogate matrix (BSA/PBS, 1 mg/mL), used for matrix effect calculation.

**Figure S1:** Representative chromatograms of the eight target amino acids and proline-d_3_ (IS), demonstrating method specificity.

Table S1. Post-preparative stability after 24 and 48h and freeze-thaw stability

| Analyte | Sample type | Autosampler stability | | Freeze-thaw stability |
| --- | --- | --- | --- | --- |
|  |  | 24h | 48h |  |
|  |  | % of initial concentration | % of initial concentration | % of initial concentration |
| Alanine | Low Cal 50 | 92.29 | 94.16 | 108.86 |
|  | High Cal 150 | 94.88 | 92.97 | 110.76 |
|  | Unspiked mix | 92.98 | 95.55 | 117.32 |
| Arginine | Low Cal 15 | 104.10 | 104.71 | 99.01 |
|  | High Cal 50 | 97.49 | 96.93 | 99.25 |
|  | Unspiked mix | 90.68 | 88.56 | 95.98 |
| Glutamic acid | Low Cal 50 | 102.68 | 106.57 | 97.59 |
|  | High Cal 150 | 96.29 | 96.45 | 95.88 |
|  | Unspiked mix | 105.58 | 106.96 | 95.38 |
| Glutamine | Low Cal 10 | 94.73 | 96.73 | 77.99 |
|  | High Cal 150 | 92.54 | 90.41 | 86.35 |
|  | Unspiked mix | 93.16 | 92.78 | 68.74 |
| Glycine | Low Cal 50 | 97.15 | 107.07 | 105.43 |
|  | High Cal 150 | 95.04 | 92.53 | 95.75 |
|  | Unspiked mix | 101.83 | 92.99 | 106.77 |
| Hydroxyproline | Low Cal 10 | 95.93 | 97.55 | 84.40 |
|  | High Cal 150 | 97.35 | 98.43 | 95.15 |
|  | Unspiked mix | 94.75 | 95.19 | 88.85 |
| Ornithine | Low Cal 15 | 107.59 | 109.06 | 97.49 |
|  | High Cal 50 | 99.39 | 100.33 | 97.28 |
|  | Unspiked mix | <LLOQ | <LLOQ | 283.38 |
| Proline | Low Cal 10 | 100.57 | 101.80 | 103.05 |
|  | High Cal 150 | 99.45 | 99.84 | 95.73 |
|  | Unspiked mix | 103.56 | 98.26 | 104.24 |

Table S2. Carry over

| No | Compound | Calculated carry-over (%) |
| --- | --- | --- |
| 1 | Alanine | No chromatographic peak of analyte in blank sample |
| 2 | Arginine | 17.33 |
| 3 | Glutamic acid | 11.66 |
| 4 | Glutamine | 9.13 |
| 5 | Glycine | 9.51 |
| 6 | Hydroxyproline | 6.60 |
| 7 | Ornithine | 82.50 |
| 8 | Proline | 6.40 |

Table S3. Calibration curve parameters used for matrix effect assessment.

| Analyte | Slope neat MeOH | r² neat | Slope BSA/PBS | r² matrix | Matrix Effect (%) |
| --- | --- | --- | --- | --- | --- |
| Alanine | 0.02057 | 0.9924 | 0.020467 | 0.9925 | 99.50 |
| Arginine | 0.454745 | 0.9900 | 0.609950 | 0.9899 | 134.13 |
| Glutamic acid | 0.088837 | 0.9953 | 0.124723 | 0.9973 | 140.40 |
| Glutamine | 0.147477 | 0.9961 | 0.188522 | 0.9067 | 127.83 |
| Glycine | 0.005075 | 0.9571 | 0.005504 | 0.9931 | 108.45 |
| Hydroxyproline | 0.209524 | 0.9941 | 0.2045 | 0.9988 | 97.62 |
| Ornithine | 0.135928 | 0.9943 | 0.164858 | 0.9957 | 121.28 |
| Proline | 1.086392 | 0.9965 | 1.251401 | 0.9993 | 115.19 |

Calibration curves were prepared in neat methanol and in surrogate matrix (BSA in PBS, 1 mg/mL). Matrix effect (%) = (slope in surrogate matrix / slope in neat methanol) × 100%. Values >100% indicate ion enhancement; values <100% indicate ion suppression; r² = coefficient of determination (weighting factor 1/x).

Figure S1. Merged, extracted ion chromatograms for all analytes of interest and internal standard (IS) – proline-d_3_.
